# DOCK1 Regulates Growth and Motility through the RRP1B-Claudin-1 Pathway in Claudin-Low Breast Cancer Cells

**DOI:** 10.3390/cancers11111762

**Published:** 2019-11-08

**Authors:** Shih-Kai Chiang, Wei-Chao Chang, Shuen-Ei Chen, Ling-Chu Chang

**Affiliations:** 1 Department of Animal Science, National Chung Hsing University, Taichung 40227, Taiwan; shihkaichiang@gmail.com; 2 Center for Molecular Medicine, China Medical University Hospital, Taichung 40447, Taiwan; proma1007@gmail.com; 3 Innovation and Development Center of Sustainable Agriculture (IDCSA), National Chung Hsing University, Taichung 40227, Taiwan; 4 The iEGG and Animal Biotechnology Center, National Chung Hsing University, Taichung 40227, Taiwan; 5 Research Center for Sustainable Energy and Nanotechnology, National Chung Hsing University, Taichung 40227, Taiwan; 6 Chinese Medicinal Research and Development Center, China Medical University Hospital, Taichung 40447, Taiwan; 7 Department of Biological Science and Technology, China Medical University, Taichung 40402, Taiwan

**Keywords:** dedicator of cytokinesis 1, claudin-1, claudin-low breast cancer, DNA methyltransferase, ribosomal RNA processing protein 1 homolog B

## Abstract

Dedicator of cytokinesis 1 (DOCK1) is a critical regulator of cancer metastasis. Claudins are transmembrane proteins that play a role in epithelial barrier integrity. Due to a loss or low expression of claudins (CLDN), the claudin-low type of triple-negative breast cancer (TNBC) is characterized by a mesenchymal-like phenotype with strong metastatic potential. In order to elucidate the mechanism of DOCK1 in cancer metastasis, we first analyzed the transcriptomic changes using a clinical database of human TNBC and found that the increase in DOCK1 expression was highly correlated with the poor survival rate of TNBC patients. Interference with DOCK1 expression by shRNA resulted in re-expression of claudin-1 in conjunction with significant inhibition of cell viability and motility of claudin-low breast cancer cells. Accordingly, overexpression of claudin-1 suppressed cell viability and migration. Genetic knockdown and pharmacological blockade of Rac1/Rac2 up-regulated claudin-1. DOCK1 knockdown also caused a decrease in DNA methyltransferase (DNMT) expression and an increase in claudin-1 transcript and promoter activity. Furthermore, RRP1B mediated DOCK1 depletion, which up-regulated claudin-1 expression, cell viability, and motility in claudin-low breast cancer cells. This study demonstrated that DOCK1 mediates growth and motility through down-regulated claudin-1 expression via the RRP1B–DNMT–claudin-1 pathway and that claudin-1 serves as an important effector in DOCK1-mediated cancer progression and metastasis in claudin-low breast cancer cells.

## 1. Introduction

Breast cancer is the second leading cause of cancer-related death in women worldwide [1]. Breast cancer is a genetically heterogeneous group of tumors and can be further subdivided into several types, including luminal estrogen receptor- and progesterone receptor-positive (ER^+^/PR^+^), human epidermal growth factor receptor 2 (HER2)-positive, and triple-negative (ER^−^/PR^−^/HER2^−^) cancers. The claudin-low subtype was newly identified within triple-negative breast cancer (TNBC), characterized by the low expression of tight junction and adherens proteins, including claudin-1, -3, -4, -7, and E-cadherin [2]. Claudin-low tumors demonstrate a mesenchymal-like phenotype and an epithelial-to-mesenchymal transition (EMT) signature, which results in more aggressive features and less favorable outcomes [3,4]. As of now, a promising therapy capable of curing aggressive TNBC is lacking, which reflects insufficient knowledge about TNBC development and progression [3]. 

EMT is a critical process in cancer cells, during which their phenotype changes from a noninvasive phenotype into an invasive one and, during this process, cancer cells become anchorage-independent and exhibit dramatically enhanced motility and aggressiveness [5,6,7]. Claudins are integral membrane proteins that connect tight junctions with cytoskeletons [8,9]. Due to the disruption of intracellular adhesion and enhanced motility, the loss of claudin-1 is associated with tumor cell invasiveness, high recurrence status, and a shorter recurrence-free survival in breast cancer [10], oral squamous cell carcinoma [11], and gastric carcinoma patients [11]. Claudin-1 has been regarded as a tumor suppressor in breast cancer due to its frequent down-regulation during tumor progression [10,12,13,14]. Re-expression of claudin-1 induces cell apoptosis and suppresses the tumorigenic transformation of human breast epithelial cells [15,16]. Forced claudin-1 expression, therefore, could be a useful approach in TNBC treatment, especially for the claudin-low subtype. 

**Figure 1:**
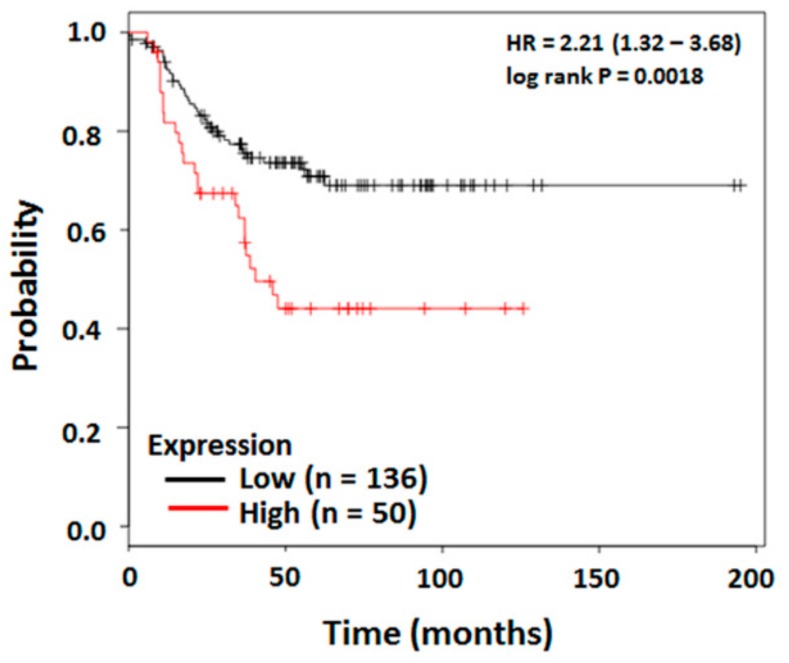
The negative correlation between DOCK1 and survival rate in TNBC patients. The overall survival of TNBC patients with high (red) or low (black) expression of DOCK1 status is shown in Kaplan–Meier Plotter (Breast Cancer), which was obtained in www.kmplot.com. The desired Affymetrix ID is valid: 203187_at (DOCK1). The *p*-values were calculated using the log-rank test.

The dedicator of cytokinesis (DOCK) proteins belong to the atypical Rho guanine nucleotide exchange factor family and play critical roles in a variety of cellular processes, including cytoskeletal organization, cell adhesion, migration [17], and phagocytosis [18]. DOCK1 has been shown to function in cell motility, myoblast fusion, and Rac1-mediated phagocytosis [18]. Moreover, DOCK1 mediates EGFRvIII stimulation in glioblastoma tumorigenesis [19], as well as in HER2-accelerated breast cancer progression [20]. More importantly, a high level of DOCK1 expression is associated with poor prognosis in patients with basal-like breast cancer and ovarian cancer [20,21], which suggests an operative machinery by DOCK1 in the tumorigenesis and metastasis of TNBC. However, the physiological functions of DOCK1 and its mechanisms in the tumorigenesis and metastasis of TNBC remain elusive. Transcriptomic investigation of the clinical database of human TNBC showed a high correlation between DOCK1 expression and poor survival rate of TNBC patients (Figure 1). We hypothesized that DOCK1 might associate with proliferation and motility of TNBC. The present study aimed to elucidate the underlying mechanisms of DOCK1-regulated metastasis in claudin-low breast cancer (CLBC). 

## 2. Results

### 2.1. DOCK1 Transcript Level is Negatively Correlation to Survival Rate of TNBC Patients

We first investigated the correlation between the DOCK1 transcript level and the probability of survival of TNBC patients using the dataset from the Kaplan–Meier Plotter (Breast Cancer). Survival curves of TNBC patients were restricted with ER-negative, PR-negative, and HER2-negative conditions. The results showed that overall survival declined significantly in patients whose tumors overexpressed DOCK1 (N = 186, log-rank test *p* = 0.0018, HR = 2.21) (Figure 1), which suggests that TNBC patients with higher DOCK1 expression have a shorter longevity.

### 2.2. DOCK1 is Involved in the Growth and Motility of CLBC Cells

**Figure 2:**
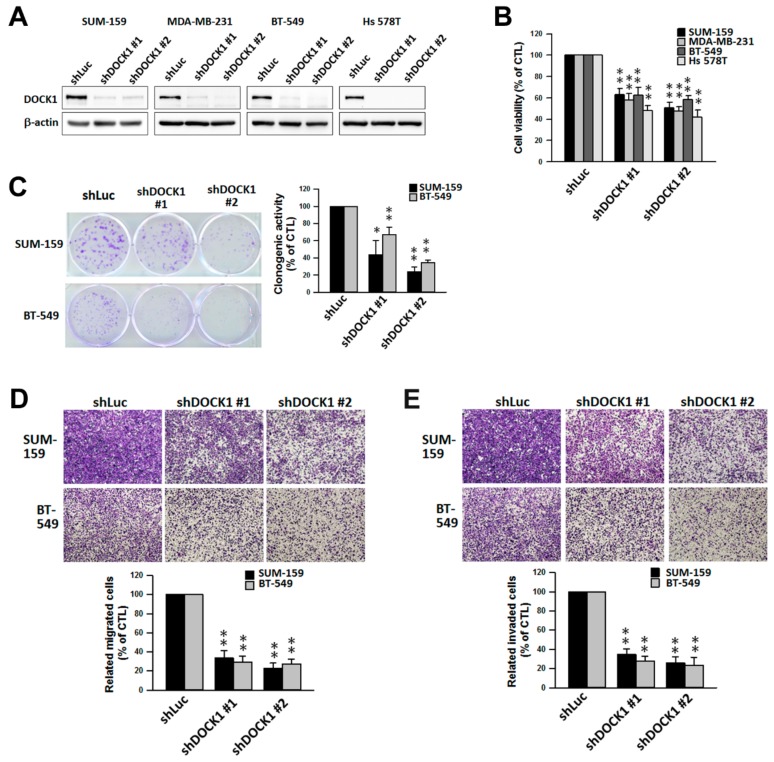
Genetic knockdown of DOCK1 suppresses cell growth and motility of claudin-low breast cancer cells. Claudin-low breast cancer cells were treated with specific shRNA against DOCK1 (shDOCK1) for three days. Depletion of DOCK1 by shDOCK1 (**A**) inhibited cell viability (**B**), clonogenic activity (**C**), migration (**D**), and invasion (**E**). The results are expressed as the mean ± SE from three independent experiments. * *p* < 0.05; ** *p* < 0.01, compared with the control group (shLuc).

The role of DOCK1 in cell growth and motility of CLBC cells was next investigated using a gene silencing approach with short hairpin RNA (shRNA) in four CLBC lines: SUM-159, MDA-MB-231, BT-549, and Hs 578T [2]. Treatment with shDOCK1 depleted cellular DOCK1 levels (Figure 2A) and significantly suppressed cell viability, and clonogenic activity, migration, and invasion (Figure 2B–E), which suggests the involvement of DOCK1 in the growth and motility of CLBC cells. 

### 2.3. Knockdown of DOCK1 Rescues the Expression of Claudin-1 in CLBC Cells

**Figure 3:**
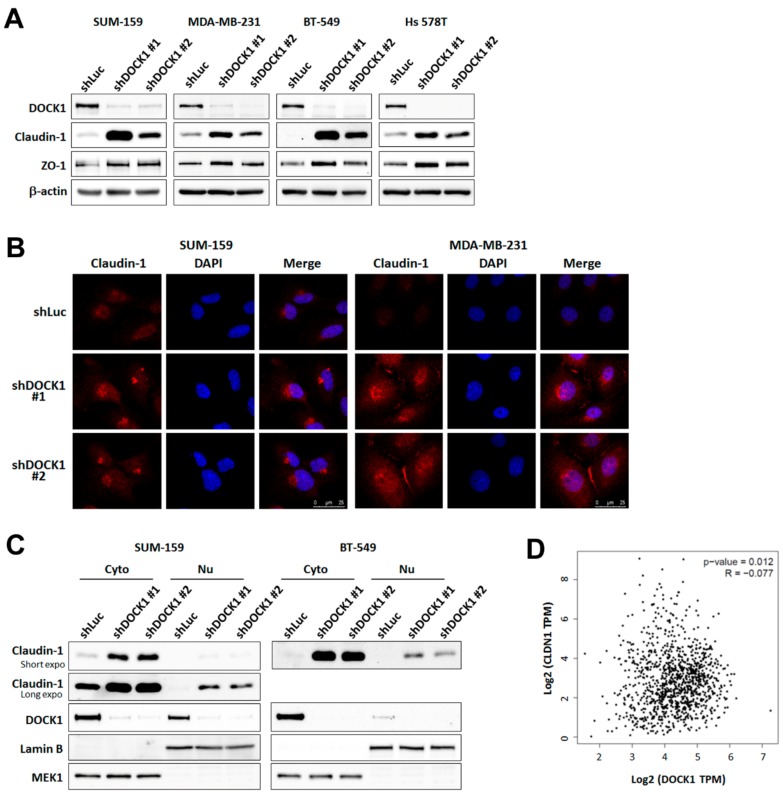
Knockdown of DOCK1 up-regulates the expression of claudin-1 in claudin-low breast cancer cells. Claudin-low breast cancer cells were treated with the shDOCK1 for three days. Cells were lysed or separated into cytosolic (Cyto) and nuclear (Nu) fractions. Claudin-1 levels were determined by Western blot analysis (**A**,**C**) and immunofluorescence staining (**B**), and were imaged by a confocal microscopy at 400× magnification. Scale bar = 25 μm. Representative images from three independent experiments are shown. (**D**) Correlation between DOCK1 and claudin-1 expression in TNBC patients was analyzed through Gene Expression Profiling Interactive Analysis (GEPIA) (http://gepia.cancer-pku.cn/).

EMT-related proteins, including Snail, Slug, vimentin, Twist1/2, E-cadherin, N-cadherin, α-catenin, β-catenin, and ZEB1, were not affected by shDOCK1 intervention (Supplementary Figure S1). However, two tight junction components, claudin-1 and zonula occludens (ZO)-1, were significantly elevated (Figure 3A). Increased claudin-1 was distributed around the perinuclear region and nuclei of CLBC cells (Figure 3B,C). The correlation coefficient between DOCK1 and claudin-1 (encoded by the *CLDN1* gene) expression in TNBC patients according to Gene Expression Profile Interactive Analysis (GEPIA) was −0.077, *p* = 0.012 (Figure 3D), which demonstrates a significant negative correlation. These results validate the role of DOCK1 in regulating claudin-1 expression in clinical cases of TNBC.

### 2.4. Claudin-1 Mediates DOCK1-Regulated Viability and Motility of CLBC Cells

**Figure 4:**
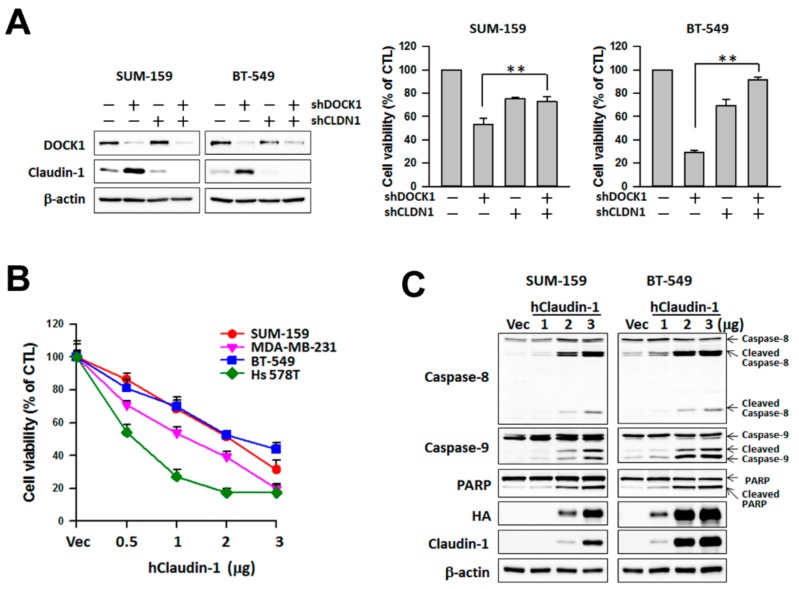
Up-regulation of claudin-1 mediates cell viability suppressed by DOCK1 deletion in claudin-low breast cells. (**A**) The knockdown of claudin-1 attenuates the shDOCK1-inhibited cell viability. Claudin-low breast cancer cells were treated with shDOCK1 and/or sh*CLDN1* for three days. Cells were harvested to measure protein expression and cell viability. The results are expressed as the mean ± SE from three independent experiments. ** *p* < 0.01, compared with the control group. Cell viability (**B**) and protein expression (**C**) were assessed at 48 h after transfection with the human claudin-1-expressed plasmid (hClaudin-1) or the control vector (Vec).

In order to investigate whether the elevation of claudin-1 plays a critical role in the decrease of DOCK1 depletion-modulated cell viability and motility, claudin-1 knockdown was performed. Treatment with specific claudin-1 shRNA (sh*CLDN1*) significantly rescued cell viability inhibited by DOCK1 depletion (Figure 4A), while forced expression of claudin-1 inhibited cell viability and induced caspase activation, including that of caspase-8, -9, and PARP in a dose-dependent manner in CLBC (Figure 4B,C). The results suggest that up-regulation of claudin-1 expression mediates the effect of DOCK1 depletion on the cell viability of CLBC cells. 

### 2.5. Rac1 and Rac2 Mediate DOCK1 Depletion-Induced Up-Regulation of Claudin-1 Expression

**Figure 5:**
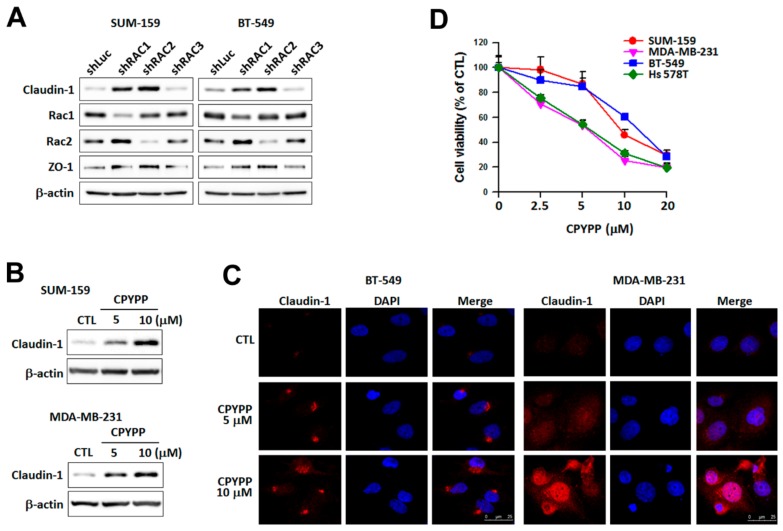
Rac1 and Rac2 mediate the up-regulation of claudin-1 by DOCK1 depletion. Claudin-low breast cancer cells were treated with specific shRNAs against Rac1, Rac2, and Rac3 for three days. The collected cells were used to determine protein expression (**A**). Cells treated with various levels of CPYPP for 24 h were used to determine claudin-1 expression by Western blot analysis (**B**) and immunofluorescence staining, as imaged by confocal microscopy at 400× magnification; scale bar = 25 μm (**C**) and cell viability analysis (**D**).

Whether Rac plays a role in DOCK1-increased claudin-1 was examined with specific shRNA of Rac. Treatment of shRAC1 and shRAC2, but not shRAC3, resulted in the re-expression of claudin-1 in CLBC cells (Figure 5A). Claudin-1 expression, as well as cell viability inhibition, were also elevated by CPYPP, a pharmacological inhibitor of DOCK1, which binds to the DHR-2 domain of DOCK1 to disrupt the interaction with Rac1 [22] (Figure 5B–D). Increased claudin-1 was distributed in the perinuclear and nuclear regions (Figure 5C). Accordingly, Rac1 and Rac2 act downstream of DOCK1 depletion in regulating claudin-1 expression.

### 2.6. DOCK1 Depletion Increases Claudin-1 Transcripts

**Figure 6:**
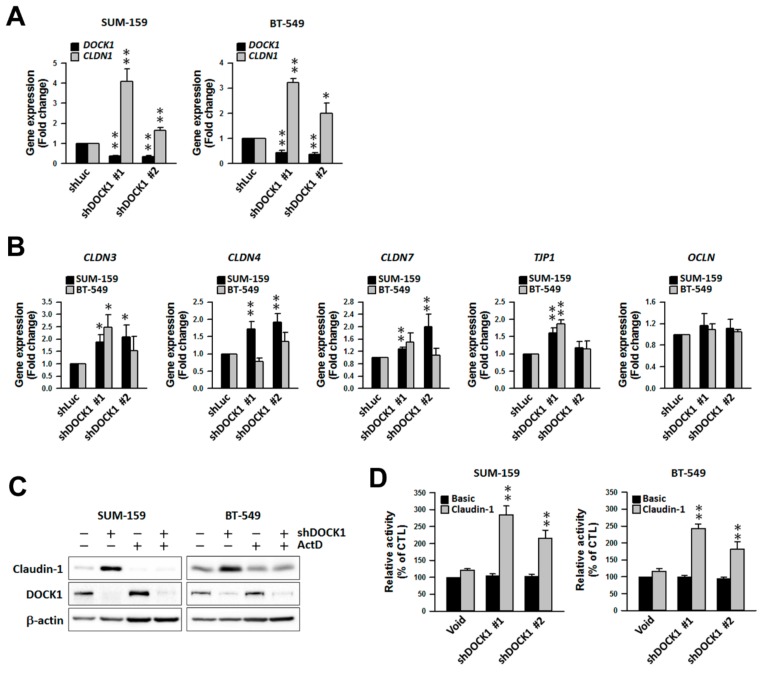
Depletion of DOCK1 transcriptionally upregulates the CLDN and tight-junction protein gene expression. (**A**,**B**) Claudin-low breast cancer cells were treated with shDOCK1 for three days. Collected cells were used for gene expression analysis by qRT-PCR analysis. The levels shown by qRT-PCR analysis were normalized to the level of β-actin (*ACTB*) and were expressed as the mean ± SE of three independent experiments. * *p* < 0.05; ** *p* < 0.01, compared with the control (shLuc). (**C**) Claudin-low breast cancer cells treated with shDOCK1 for 24 h and then with 1 μM actinomycin D (ActD) for 48 h were used for Western blot analysis. (**D**) Claudin-low breast cancer cells transfected with the pGL4-Basic or pGL4-Claudin-1 vector for 24 h and then with shDOCK1 for two days were used for the promoter activity assay. ** *p* < 0.01, compared with the control (Void).

Results of qRT-PCR analysis showed that claudin-1 transcript (*CLDN1*) levels were significantly increased by shDOCK1 intervention (Figure 6A), while moderate increases were observed in *CLDN3* (encoded claudin-3), *CLDN4* (encoded claudin-4), and *CLDN7* (encoded claudin-7) expression (Figure 6B). *TJP1* (encoded ZO-1) was also increased, whereas *OCLN* (encoded occludin), remained unchanged (Figure 6B). Furthermore, treatment with the transcription inhibitor actinomycin D significantly attenuated the increase in claudin-1 expression by shDOCK1 (Figure 6C), which is consistent with the promoter activity of claudin-1 by DOCK1 depletion (Figure 6D). This suggests an operative machinery by DOCK1 depletion specific to claudin-1 expression that functions by regulating its promoter activity, which leads to a decrease in the cell viability and motility of CLBC cells. 

### 2.7. DOCK1 Depletion Increases DNA Demethylation of the CLDN1 Promoter

**Figure 7:**
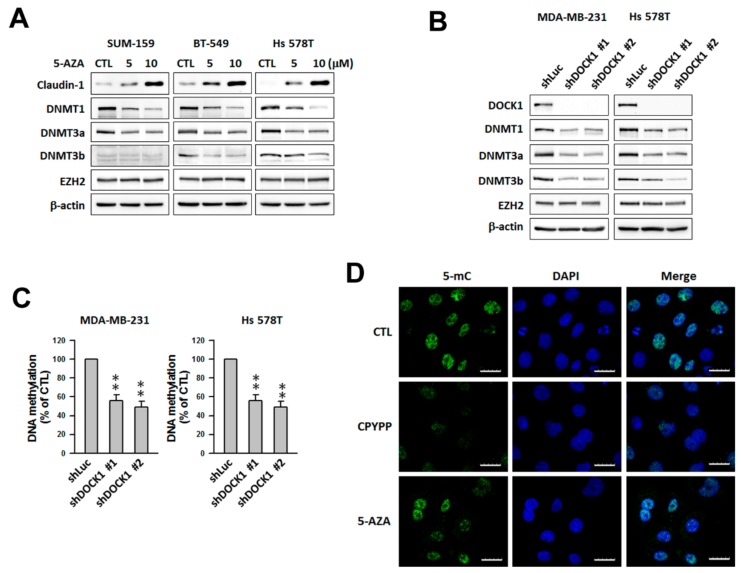
Up-regulation of claudin-1 expression by DOCK1 depletion is mediated through down-regulation of DNMTs and DNA methylation status. (**A**–**C**) The inhibition of DNMTs increased the expression of claudin-1. Claudin-low breast cancer cells treated with various levels of 5-azacytidine (5-AZA) for 24 h (**A**) or with shDOCK1 for three days (**B**,**C**) were used for protein determination (**A**,**B**) or DNA methylation assay (**C**). ** *p* < 0.01, compared with the control (shLuc). (**D**) SUM-159 cells treated with 5 μM CPYPP or 10 μM 5-AZA for 24 h were used for DNA methylation analysis. DNA methylation was imaged after immunostaining using an anti-5-methylcytosine (5-mC) antibody under a confocal microscope at 400× magnification. Scale bar = 25 μm.

Treatment with a specific DNA methyltransferase inhibitor, 5-azacytidine (5-AZA), led to a significant increase in claudin-1 protein abundance (Figure 7A). Genetic knockdown of DOCK1 suppressed DNA methyltransferase 1 (DNMT1), DNMT3a, and DNMT3b expression (Figure 7B), which is consistent with a decrease in global DNA methylation (Figure 7C). Immunofluorescence staining with an anti-5-methylcytosine (5-mC) antibody further confirmed the results showing that CPYPP, a DOCK1 inhibitor, also inhibited global DNA methylation (Figure 7D). These data implicated DOCK1 in affecting claudin-1 expression via regulation of DNA methylation status in the promoter region. 

### 2.8. DOCK1 Regulates Claudin-1 through RRP1B and c-Jun

**Figure 8:**
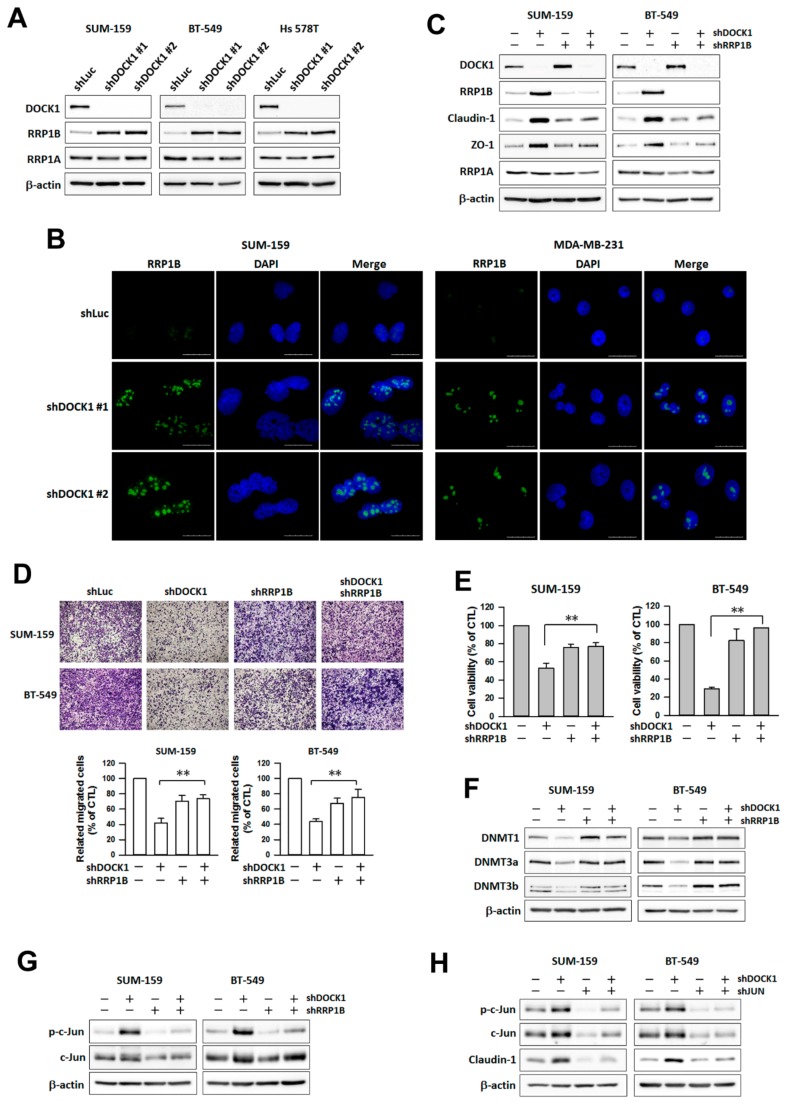
RRP1B mediates the down-regulation of DNMTs, the up-regulation of claudin-1, and increased cell viability caused by DOCK1 depletion. (**A**,**B**) Claudin-low breast cancer cells were treated with the shDOCK1 for three days. Cancer cells were collected for protein determination (**A**) or immunofluorescence staining (**B**). (**B**) RRP1B was stained with a specific anti-RRP1B antibody. Nuclei were counterstained with DAPI. Magnification, 400×. Scale bar = 25 μm. (**C**–**H**) Claudin-low breast cancer cells were treated with the shDOCK1, shRRP1B, or shJUN for three days followed by the protein determination (**C**,**F**–**H**), cell migration (**D**), or cell viability (**E**). The results are expressed as the mean ± SE from three independent experiments. * *p* < 0.05; ** *p* < 0.01, compared with the control (shLuc).

To verify the possible pathway involved in claudin-1 up-regulation induced by DOCK1 depletion, liquid chromatography-mass spectrometry was performed to investigate the proteomic alterations that occurred as a result of DOCK1 depletion. The results showed that ribosomal RNA processing protein 1B (RRP1B), a metastatic modulator in breast cancer [23,24], exhibited the most changes (up to 15-fold) after a three day-knockdown of DOCK1 in BT-549 cells (data not shown). DOCK1 depletion significantly increased RRP1B levels, whereas the other protein, ribosomal RNA processing protein 1A (RRP1A), remained unchanged (Figure 8A). Increased RRP1B was mainly distributed in the nuclei (Figure 8B and Supplementary Figure S2). Genetic knockdown of RRP1B attenuated the increase in claudin-1 and ZO-1 expression (Figure 8C), cell migration (Figure 8D), cell viability (Figure 8E), and caspase activation (Supplementary Figure S3), and concomitantly rescued DNMT expression induced by DOCK1 depletion (Figure 8F). 

In the phospho-kinase array analysis, DOCK1 knockdown significantly altered the phosphorylation status of specific proteins, of which c-Jun was considerably activated (Supplementary Figure S4). The phosphorylation of c-Jun, increased by DOCK1 depletion, was significantly reversed by RRP1B knockdown (Figure 8G). Furthermore, shJUN intervention attenuated claudin-1 expression induced by DOCK1 depletion (Figure 8H). These results suggest that RRP1B responds to DOCK1 depletion and mediates downstream DNMT-Claudin-1 transcription, which ultimately impairs the cell growth and migration of CLBC cells.

## 3. Discussion

DOCK1 is highly expressed in malignant breast cancer cells, especially in TNBC [25]. Our data showed that DOCK1 depletion induced claudin-1 re-expression, and a negative correlation between DOCK1 and claudin-1 has been observed in clinical TNBC patients, which indicates that claudin-1 might be negatively regulated by DOCK1. Claudin-1 knockdown ameliorated DOCK1-mediated cell growth inhibition and migration, which demonstrates that DOCK1-regulated claudin-1 plays an important role in CLBC growth. It may also explain the high aggressiveness of CLBC results from high DOCK1 expression. The DOCK1 depletion-induced increase in claudin-1 inhibited cell migration and induced caspase activation and cell death, which is consistent with the previous finding that re-expression of claudin-1 causes cell apoptosis in TNBC [15,16] and modulates the mobility of melanoma cells [26]. However, apoptosis regulation-relevant molecules modulated by claudin-1 in CLBC require further investigation. In lung adenocarcinoma, claudin-1 re-expression-modulated cell growth is associated with up-regulation of cancer invasion/metastasis suppressors (e.g., connective tissue growth factor, thrombospondin 1, deleted in liver cancer 1, occludin, and ZO-1) and down-regulation of invasion/metastasis enhancers (e.g., secreted phosphoprotein 1, cut-like homeobox 1, transforming growth factor alpha, solute carrier family 2 member 3, and placental growth factor) [27]. Other members of the claudin family have also been reported to function as tumor suppressors. Overexpression of claudin-4 significantly reduces the invasive potential and clonogenic activity, as well as pulmonary metastasis, in human pancreatic cancer cells [28], breast cancer cells [29], and clear cell renal cell carcinoma [30]. Genes other than *CLDN1*, *CLDN3*, *CLDN4*, *CLDN7*, and *ZO1* may also be coordinately regulated by DOCK1 and may contribute to tumor growth, but this remains to be investigated.

A high level of DNMT expression leads to DNA hypermethylation and contributes to oncogenic activation [31]. Dysregulation of claudin expression has been shown to be associated with DNA hypermethylation status of the promoter region of certain genes [29,30,32,33,34]. Our data showed that DOCK1 depletion decreased DNMT1, DNMT3a, and DNMT3b, as well as global DNA methylation followed by claudin-1 up-regulation in CLBC. Regulation of the methylation status by DNMT1 to induce claudin-1 expression was also observed in podocytes of streptozotocin-induced diabetic and obese (*db*/*db*) mice [35]. Other claudins can also be regulated by DNMT1. In MCF-7 and SKBR-3 breast cancer cells, DNA hypermethylation is associated with low expression of claudin-6. Inhibition of DNMT1 by 5-AZA, a DNA methyltransferase inhibitor, reverses claudin-6 expression [29]. Thereby, it could be reasoned that DOCK1 depletion also increases *CLDN3*, *CLDN4*, and *CLDN7* expression. Other methyltransferases have been identified to play a role in claudin expression. Genetic knockdown of EHMT2, a histone methyltransferase, reduces cancer cell migration and invasion by regulating the expression of EMT-related markers (E-cadherin, claudin-1, and vimentin) [36]. EZH2 regulates claudin-23 expression in colorectal cancer [37]. Our data showed that EZH2 remains unchanged in DOCK1 depletion. Meanwhile, EZH2 inhibitor GSK126 was unable to induce claudin-1 re-expression (data not shown), suggesting EZH2 was not involved in DOCK1 depletion-increased clandin-1. The DOCK1 inhibitor CPYPP also inhibits DNA methylation, which indicates the association of DOCK1 with intracellular methylation status. 

RRP1B is a nuclear protein that has been identified as a breast cancer metastasis suppressor through heterochromatinization at specific loci with hypermethylation at histone 3, which represses the expression of certain genes [23]. Overexpression of RRP1B in MDA-MB-231 cells could decrease pulmonary metastasis after injection into mice [24]. Our study showed that RRP1B was significantly up-regulated by DOCK1 depletion and that RRP1B knockdown rescued DOCK1 depletion-regulated events, which means that RRP1B mediates the effects of DOCK1. Observation by confocal microscopy showed that the increased RRP1B was located within the nucleolus. Previous studies found that RRP1B might localize to the nucleolus, where it binds to various nuclear proteins, such as nucleolin and nucleophosmin 1 [38,39]. Nucleolin and nucleophosmin 1 remain unchanged in DOCK1-depleted CLBC (data not shown). Alterations in cell adhesion induced by RRP1B suppressed tumor growth and metastasis and are associated with the expression of extracellular matrix genes and SPIA1 (serpin peptidase inhibitor, clade A, member 1) [40]. Here, we demonstrated that changes in c-Jun and DNMT expression by RRP1B mediated the up-regulation of claudin-1 by DOCK1 depletion, which led to the suppression of cell viability and invasiveness of CLBC cells.

The majority of DOCK1 is located in the cytoplasm, where it forms a complex with ELMO and acts as a guanine nucleotide exchange factor for Rac [41]. However, DOCK1 is also distributed in the nuclear complex with ELMO [42]. Herein, we found that DOCK1 is distributed in both the cytoplasmic and nuclear compartments. DOCK1 depletion increased claudin-1 expression in both the cytoplasm and nuclei. Despite the fact that tight junction proteins (claudins, ZOs, and occludin) are the most apical cell–cell contact points and are important in barrier function in epithelial and endothelial cells [43], in invasive cancer cells, claudins are abnormally distributed in the nucleus, where they mediate cell proliferation and transcription events [44,45]. Nuclear localization of claudin-1 induces structural and functional changes in EMT markers in colon cancer [44]. The nuclear localization of claudin-2 and claudin-6 has been linked to enhanced cell proliferation via promotion of cell cycle progression in lung adenocarcinoma cells [45]. We proposed that nuclear claudin-1 might interact with the genes/proteins involved in apoptosis and motility. 

**Figure 9:**
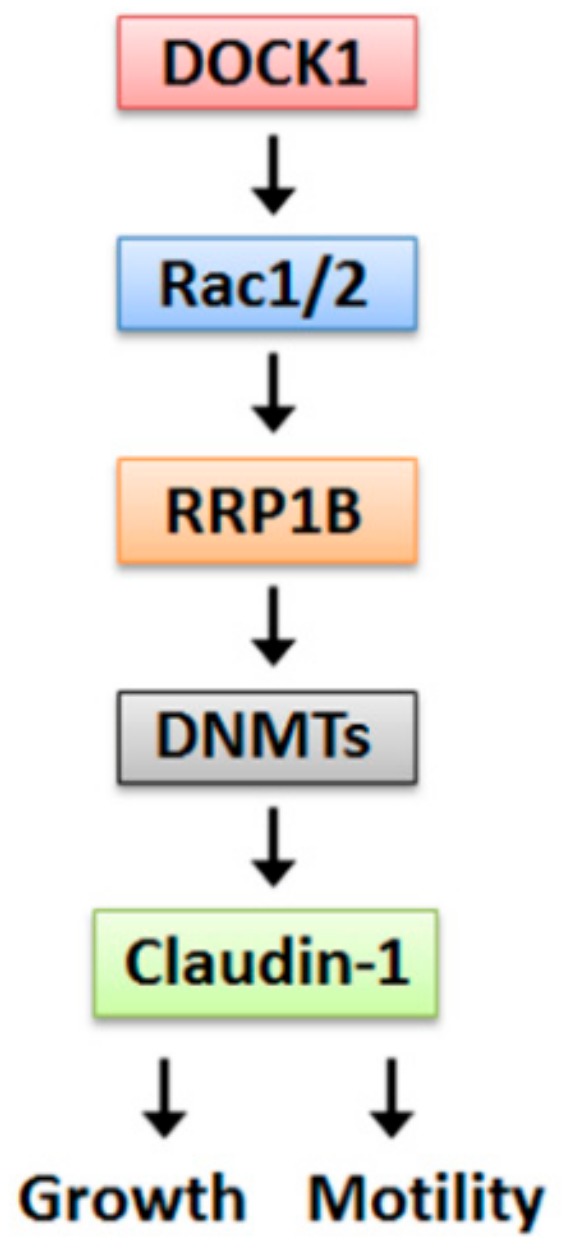
A proposed pathway of DOCK1 in the regulation of growth and motility of CLBC cells.

DOCK1 is a specific Rac-nucleotide exchange factor that regulates Rac signaling with respect to cell motility and proliferation [21,46]. Results from gene silencing and experiments involving the pharmacological inhibitor CPYPP clearly demonstrated that Rac1/2 mediate the up-regulation of claudin-1 and suppressed cell viability under conditions of DOCK1 depletion. However, the operative machinery by which DOCK1/Rac regulates RRP1B requires further investigation. Results from the phospho-kinase array analysis showed that DOCK1 knockdown altered FAK, RSK, p53, ERK, and WNK1 expression (Supplementary Figure S4), which may provide some clues for further studies. Inactivation of the TGFβ/SMAD pathway has been shown to inhibit cell invasion by suppressing DNMT1 and claudin-6 methylation and expression in MCF-7 and SKBR-3 breast cancer cell lines [29]. Our findings demonstrated that DOCK1 regulates the growth and motility capacities of CLBC cells by down-regulating claudin-1 expression through the Rac1/2-RRP1B-DNMTs cascade (Figure 9). Elucidation of oncogenic DOCK1-claudin-1 signaling might be beneficial for the development of a promising strategy against CLBC. 

## 4. Materials and Methods

### 4.1. Reagents and Antibodies 

DMEM/F12 medium, RPMI-1640 medium, fetal bovine serum (FBS), and penicillin/streptomycin were purchased from Thermo Fisher Scientific Inc. (Pittsburgh, PA, USA). CPYPP was purchased from Tocris Bioscience (Ellisville, MO, USA). Actinomycin D was purchased from Enzo Life Sciences (Farmingdale, NY, USA). 5-azacytidine (5-AZA) was obtained from Cayman Chemicals (Ann Arbor, MI, USA). Antibodies against DOCK1 (#4846), MEK1 (#8727), 5-aethylcytosine (5-mC) (#28692), HA-Tag (#3724), caspase-3 (#9662), caspase-8 (#9746), caspase-9 (#9502), PARP (#9542), vimentin (#5741), E-cadherin (#3195), N-cadherin (#4061), ZO-1 (#5406), ZEB1 (#3396), Snail (#3879), Slug (#9585), α-catenin (#3240), β-catenin (#9582), phospho-c-Jun (#9164), and c-Jun (#9165) were purchased from Cell Signaling Technology (Beverly, MA, USA). The antibody against β-actin (MAB1501) was obtained from EMD Millipore Corporation (Billerica, MA, USA). Antibodies against claudin-1 (sc-137121), DNMT3a (sc-20703), DNMT3b (sc-81252), RRP1A (sc-398970), and RRP1B (sc-398162) were purchased from Santa Cruz Biotechnology (Santa Cruz, CA, USA). Antibodies against DNMT1 (#612618) and EZH2 (#612666) were obtained from BD Biosciences (San Jose, CA, USA). The antibody that recognizes Twist1/2 (GTX127310) was purchased from GeneTex (Irvine, CA, USA). The application of antibodies used in this study is listed in the Supplementary Materials and Methods (Supplementary Table S1). Other reagents were purchased from Sigma-Aldrich (St. Louis, MO, USA). 

### 4.2. Cell Culture

All cell lines used in this study were obtained from the American Type Culture Collection (Manassas, VA, USA). SUM-159, MDA-MB-231, and Hs 578T cells were cultured in DMEM/F12 medium (Invitrogen) supplemented with 10% (*v*/*v*) FBS, 100 U/mL penicillin, and 100 μg/mL streptomycin; an additional 1 μg/mL of hydrocortisone was supplemented in the culture medium of SUM-159 cells. BT-549 cells were cultured in RPMI-1640 medium (Invitrogen) supplemented with 10% (*v*/*v*) FBS, 2 mM L-glutamine, 100 U/mL penicillin, and 100 μg/mL streptomycin. All cells were maintained at 37 °C in a humidified incubator containing 5% CO_2_. 

### 4.3. Gene Knockdown by shRNA

The pCMV-ΔR8.91, pMD.G, and specific short hairpin PLKO.1 plasmids were purchased from the National RNAi Core Facility Academia Sinica (Taiwan). The shRNA clones used in this study are described in the Supplementary Materials and Methods (Table S2). The 293T cells were transiently transfected with the specific shRNA and packaging vectors (pCMV-ΔR8.91 and pMD.G) using Lipofectamine 2000 as a transfection reagent (Invitrogen). Forty-eight hours after transduction, lentiviral particles in the medium were filtered with a 0.22 μm filter and used for infection. Cancer cells were infected with the specific shRNA virus-containing supernatant in the presence of polybrene (8 μg/mL). Twenty-four hours after infection, the medium was replaced with normal medium supplemented with 2 μg/mL of puromycin. Cells continued to grow in the presence of puromycin for another 72 h and were then subjected to further testing.

### 4.4. CLDN1 Overexpression

Cells were cultured in a culture dish or a culture plate and allowed to attach overnight. The pCMV3-C-HA vector (Sino Biological) or the vector containing human CLDN1 cDNA (pCMV3-CLDN1-HA, Sino Biological) was transfected into the cells for 24 h. 

### 4.5. Isolation of Cytosolic and Nuclear Proteins 

After treatments, the cells were washed with phosphate-buffered saline (PBS) and detached with trypsin. Cells were then gently suspended in a hypotonic buffer (10 mM HEPES, pH 7.9, 1.5 mM MgCl_2_, 1 mM PMSF, 10 μg/mL each of leupeptin, aprotinin, pepstatin A, 1 mM Na_3_VO_4_, and 1 mM NaF) and kept on ice for 15 min. After the addition of 0.5% NP-40, the cell suspension was vigorously vortexed for 10 seconds to allow lysis and release of the cytoplasm. The cytoplasmic components (supernatants) and nuclear fraction (pellets) were separated by centrifugation at 10,000 × *g* for 2 min at 4 °C. For the purification of nuclear proteins, the nuclear pellets were washed three times in hypotonic buffer and suspended in PBS containing protease inhibitors and phosphatase inhibitors. The isolated fractions were stored at −80°C for further analysis. 

### 4.6. Cell Viability Assay

Cell viability was determined by the MTT (3-(4,5-dimethylthiazol-2-yl)-2,5-diphenyltetrazolium bromide) reduction method. After treatment with short hairpin RNA (shRNA) or other compounds, MTT solution (2 mg/mL) was added to the cell cultures, which were allowed to incubate for another 2 h. The formazan product was dissolved in DMSO and was detected spectrophotometrically at a wavelength of 570 nm with normalization by 630 nm. 

### 4.7. Colony Formation Assay 

Five hundred cells per well were seeded onto 6-well plates and maintained at 37 °C in an incubator with 5% CO_2_. Cells were treated with shDOCK1 for 24 h, after which the medium was replaced with normal culture medium containing 2 μg/mL puromycin, and the cells were maintained in culture for 1 week. Colonies were then fixed in formaldehyde (3.7%, *v*/*v*) and stained with crystal violet (0.5%, w/v). Colony formation was imaged and quantified spectrophotometrically at 595 nm after extraction in 10% acetic acid. 

### 4.8. Quantification of DNA Methylation 

Genomic DNA was extracted using the genomic DNA extraction kit (Real Genomics, Taipei, Taiwan), followed by Nuclease S1 (New England Biolabs, Ipswich, MA, USA) and Antarctic phosphatase (New England Biolabs) digestion. The methylated fractions of DNA were detected using a DNA methylation kit (Cat. #589324, Cayman). 

### 4.9. Immunofluorescence Staining 

Cells were grown on sterile coverslips embedded in a 6-well plate. After treatments, cells were fixed in 3.7% (w/v) formaldehyde followed by permeabilization with 0.2% (*v*/*v*) Triton X-100. After blocking with 2% (w/v) bovine serum albumin (BSA) in PBS, cells were stained with the specific antibodies and then reacted with Alexa Fluor^®^ 488- or Alexa Fluor^®^ 594-conjugated secondary antibodies (Invitrogen). Coverslips were mounted (DAPI Fluoromount-G, Cat. #0100-20, SouthernBiotech), and fluorescence images were obtained on a Leica Microsystems TCS SP8 Confocal Spectral microscope (Leica Microsystems, Wetzlar, Germany). 

### 4.10. Immunofluorescence Staining of 5-mC

Cells were fixed in ice-cold 70% ethanol for 5 min and then 1.5 M HCl was added, after which the cells were allowed to incubate for 30 min at room temperature. After blocking in 2% BSA-PBS, the cells were stained with the 5-mC antibody and then reacted with the Alexa Fluor^®^ 488-conjugated secondary antibody.

### 4.11. Quantitative Real-Time Reverse Transcription PCR (qRT-PCR) 

Total RNA was extracted using TRIzol^®^ Reagent (ThermoFisher). A quantity of 5 μg of total RNA was used as the template for cDNA strain synthesis using M-MLV reverse transcriptase (ThermoFisher) according to the manufacturer’s instructions. qRT-PCR analysis was performed in a LightCycler^®^ 480 II RTPCR system (Roche Applied Sciences, Manheim, Germany) using KAPA SYBR FAST qPCR Master Mix (Kapa Biosystems). Primer information is shown in the Supplementary Materials and Methods, Table S3. The expression levels of mRNA were normalized against the level of β-actin mRNA (*ACTB*) in the same samples. 

### 4.12. Migration and Invasion Assay

Cells suspended in serum-free medium (150-μL volume, 25,000 cells) were placed on top of a Transwell insert (8 μm pore size, Cat. # MCEP24H48, BD Biosciences). The lower chamber was loaded with serum-containing complete medium (700 μL per well). After incubation, cells on the Transwell filters were fixed in 4% formaldehyde and stained with 0.1% crystal violet. The migrated cells were imaged under a microscope and then extracted with 10% acetic acid for spectrophotometric quantification at 595 nm. For the invasion assay, Transwell filters were precoated with 50 μL of 1 μg/mL of Matrigel™ (Cat. # 354234, BD Bioscience) at 37 °C for 1 h. The percentages of related migrated cells were calculated by normalizing the values to cell viability in the samples. 

### 4.13. Luciferase Reporter Gene Assay 

The human claudin-1 promoter-driven luciferase construct, pGL4-Claudin-1, was obtained from Addgene (#46387). The control vector pGL4-Basic was purchased from Promega (Madison, WI, USA). Promoter activity was determined with the dual luciferase reporter assay system (Promega). Briefly, the cells were transiently transfected with the pGL4-Basic or pGL4-Claudin-1 plasmids using Lipofectamine 2000 reagent (Invitrogen). Luciferase activities were then measured 48 h after transfection and normalization with protein abundance. 

### 4.14. Western Blot Analysis

Detached cells were sonicated in PBS containing protease inhibitors (1 mM PMSF, 10 μg/mL each of leupeptin, aprotinin, and pepstatin A) and phosphatase inhibitors (1 mM Na_3_VO_4_, 1 mM NaF) and used for protein extraction with RIPA buffer. Protein concentrations were measured using a Bradford assay purchased from Bio-Rad (Hercules, CA, USA). The lysates were separated by SDS-PAGE gels and transferred onto FluoroTrans^®^ PVDF Transfer Membranes (Pall Corporation). The membranes were then blocked with Tris-buffered saline with 0.1% Tween 20 and 5% skimmed milk, followed by incubation with the primary antibodies. Membranes were then reacted with horseradish peroxidase-conjugated secondary antibodies (EMD Millipore). Signaling was visualized using the Clarity™ Western ECL Substrate (Bio-Rad) and quantified by the luminescence image analyzer ImageQuant LAS 4000 (GE Healthcare Life Sciences). 

### 4.15. Kaplan–Meier Analyses and Gene Correlation Assay 

The correlation between the survival rate of breast cancer patients and DOCK1 gene expression was analyzed using an online database, Kaplan–Meier Plotter (Breast Cancer) (http://kmplot.com/analysis/). Briefly, DOCK1 was entered into the database (http://kmplot.com/analysis/index.php?p=service&cancer=breast) to obtain Kaplan–Meier survival plots containing patients with grade 3 tumors; the search was then further restricted according to ER-, PR-, and Her2-status. The number-at-risk was indicated below the main plot. Hazard ratio (HR), 95% confidence intervals, and log-rank *p* were calculated and displayed on the webpage. *P* values < 0.05 were considered statistically significant. Gene Expression Profiling Interactive Analysis (GEPIA) (http://gepia.cancer-pku.cn/) was used to investigate the correlation between *DOCK1* expression and *CLDN1* (encodes claudin-1) in TNBC. 

### 4.16. Statistical Analysis

All data are expressed as the mean ± standard error (SE) of three independent experiments, unless stated otherwise. The data were analyzed by Student’s *t*-tests. *P* values < 0.05 were considered statistically significant.

## 5. Conclusions

In CLBC cells, DOCK1 mediates cell growth and motility through down-regulating the expression of claudin-1 via the RRP1B–DNMT–claudin-1 pathway, and claudin-1 serves as an important effector in DOCK1-mediated cancer progression and metastasis.

## Supplementary Materials

The following are available online at https://www.mdpi.com/2072-6694/11/11/1762/s1, Table S1: Antibody information. Table S2: Primer information in qRT-PCR analysis. Table S3: shRNAs information in gene knockdown analysis. Figure S1: Effect of DOCK1 depletion on EMT-related protein expression. Figure S2: Knockdown of DOCK1 increases nuclear RRP1B levels. Figure S3: Knockdown of RRP1B attenuates caspase activation by DOCK1 depletion. Figure S4: DOCK1 depletion alters the phosphorylation of specific protein kinases.
