# Supplementary material for: DOCK1 Regulates Growth and Motility through the RRP1B-Claudin-1 Pathway in Claudin-Low Breast Cancer Cells
Source: Cancers (Basel). 2019 Nov 8;11(11):1762. doi: 10.3390/cancers11111762 (PMC6896004; doi:10.3390/cancers11111762)
Supplement: Supplementary file 1 [file cancers-11-01762-s001.pdf]

Supplementary Materials and Methods

Table S1. Antibody information in this study

Antibodies	Catalog No., Company	Analyses (dilution ratio)
5-Methylcytosine (5-mC)	#28692, Cell Signaling Tech.	Immunofluorescence (1: 500)
α-Catenin	#3240, Cell Signaling Tech.	Western Blotting (1: 1000)
β-actin	MAG1501; EMD Millipore	Western Blotting (1: 10000)
β-Catenin	#9582, Cell Signaling Tech.	Western Blotting (1: 2000)
Caspase-3	#9662, Cell Signaling Tech.	Western Blotting (1: 1000)
Caspase-8	#9746, Cell Signaling Tech.	Western Blotting (1: 1000)
Caspase-9	#9502, Cell Signaling Tech.	Western Blotting (1: 1000)
c-Jun	#9165, Cell Signaling Tech.	Western Blotting (1: 1000)
Claudin-1	sc-137121, Santa Cruz Biotech.	Western Blotting (1: 1000)<br>Immunofluorescence (1: 500)
DNMT1	#61618, BD Biosciences	Western Blotting (1: 1000)
DNMT3a	sc-20703, Santa Cruz Biotech.	Western Blotting (1: 1000)
DNMT3b	sc-81252, Santa Cruz Biotech.	Western Blotting (1: 500)
DOCK1	#4846, Cell Signaling Tech.	Western Blotting (1: 1000)
E-Cadherin	#3195, Cell Signaling Tech.	Western Blotting (1: 1000)
EZH2	#612666, BD Biosciences	Western Blotting (1: 1000)
HA-Tag	#3724, Cell Signaling Tech.	Western Blotting (1: 3000)
MEK1	#8727, Cell Signaling Tech.	Western Blotting (1: 1000)
N-Cadherin	#4061, Cell Signaling Tech.	Western Blotting (1: 1000)
PARP	#9542, Cell Signaling Tech.	Western Blotting (1: 1000)
phospho-c-Jun	#9164, Cell Signaling Tech.	Western Blotting (1: 1000)
RRP1A	sc-398970, Santa Cruz Biotech.	Western Blotting (1: 1000)
RRP1B	sc-398162, Santa Cruz Biotech.	Western Blotting (1: 1000)<br>Immunofluorescence (1: 500)
Slug	#9585, Cell Signaling Tech.	Western Blotting (1: 1000)
Snail	#3879, Cell Signaling Tech.	Western Blotting (1: 1000)
Twist1/2	GTX127310, GeneTex	Western Blotting (1: 1000)
Vimentin	#5714, Cell Signaling Tech.	Western Blotting (1: 2000)
ZEB1	#3396, Cell Signaling Tech.	Western Blotting (1: 1000)
ZO-1	#5406, Cell Signaling Tech.	Western Blotting (1: 1000)
Goat anti-Mouse IgG (H+L) Highly Cross-Adsorbed Secondary Antibody, Alexa Fluor 488	A-11029, ThermoFisher Scientific	Immunofluorescence (1: 1000)<br>Assay: Intracellular location of RRP1B (Figure 8B)
Goat anti-mouse IgG (H+L) Highly Cross-Adsorbed Secondary Antibody, Alexa Fluor 594	A-11032, ThermoFisher Scientific	Immunofluorescence (1: 1000)<br>Assay: Intracellular location of Claudin-1 (Figure 3B and Figure 5C)
Rabbit IgG (H+L) Highly Cross-Adsorbed Secondary Antibody, Alexa Fluor 488	A-21206, ThermoFisher Scientific	Immunofluorescence (1: 1000)<br>Assay: 5-mC staining (Figure 7D)

Table S2. Primer information in qRT-PCR analysis.

Gene (encoded protein)	Gene Bank no.	Primer sequence
ACTB (β-actin)	AY582799.1	F: 5’-CCAACCGCGAGAAGATGA-3’<br>R: 5’-CCAGAGGCGTACAGGGATAG -3’
CLDN1 (claudin-1)	NM_021101.4	F: 5’-TTTACTCCTATGCCGGCGAC-3’<br>R: 5’-GAGGATGCCAACCACCATCA-3’
CLDN3 (claudin-3)	NM_001306.3	F: 5’-GGGACTTCTACAACCCCGTG-3’<br>R: 5’-CTTGGTGGCCGTGTACTTCT-3’
CLDN4 (claudin-4)	NM_001305.4	F: 5’- TCTCCTCTGTTCCGGGTAGG -3’<br>R: 5’- TCCCCTCTAAACCCGTCCAT -3’
CLDN7 (claudin-7)	NM_001307.5	F: 5’-TCTTTATTGGCTGGGCAGGG-3’<br>R: 5’-ACCCAGCCTTGCTCTCATTC-3’
OLDN (occludin)	AH011257.2	F: 5’-AAGGTTCCATCCGAAGCAGG-3’<br>R: 5’-GGGAGTGTAGGTGTGGGTG-3’
TJP1 (zonula occludens 1/ZO-1; tight junction protein/TJP1)	NM_003257.4	F: 5’- CGGGAAGTTACGTGGCGAAG-3’<br>R: 5’- TCGGACAAAAGTCCGGGAAG-3’

Table S3. shRNA information in gene knockdown analysis.

shRNA	Target gene	Gene ID	shRNA clone
shCLDN1 #1	*CLDN1*	9076	TRCN0000117333
shCLDN1 #2	*CLDN1*	9076	TRCN0000117334
shDOCK1 #1	*DOCK1*	1793	TRCN0000029074
shDOCK1 #2	*DOCK1*	1793	TRCN0000029076
shJUN	*JUN*	3725	TRCN0000338165
shLuc			TRCN0000231740
TRC1.Void			ASN0000000002
shRAC1	*RAC1*	5879	TRCN0000008430
shRAC2	*RAC2*	5880	TRCN0000047274
shRAC3	*RAC3*	5881	TRCN0000047290
shRRP1B #1	*RRP1B*	23076	TRCN0000131094
shRRP1B #2	*RRP1B*	23076	TRCN0000129332

Supplementary Figures

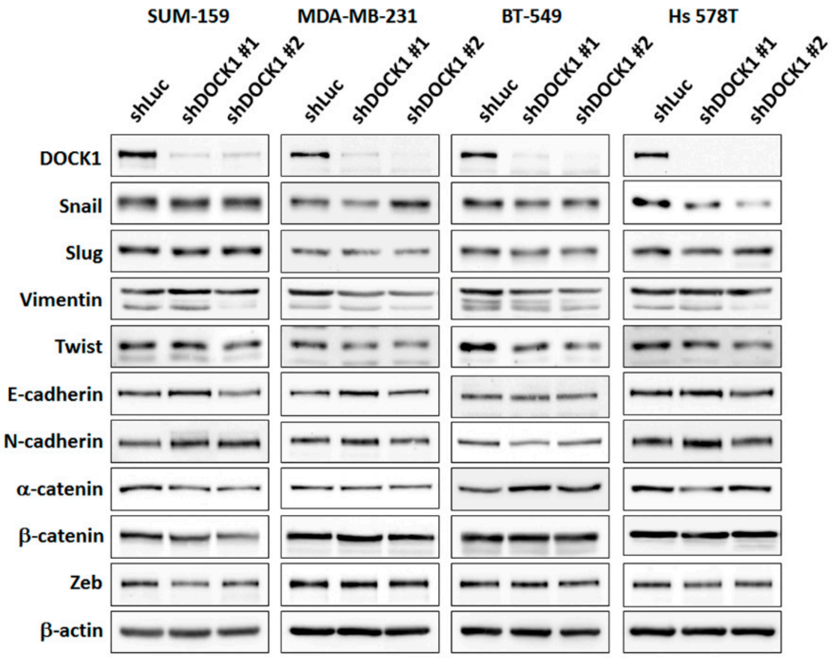

Figure S1. Effect of DOCK1 depletion on EMT-related protein expression. Claudin-low breast cancer cells were treated with shDOCK1 for 3 days. Cells were then harvested for protein abundance analysis by Western blot.

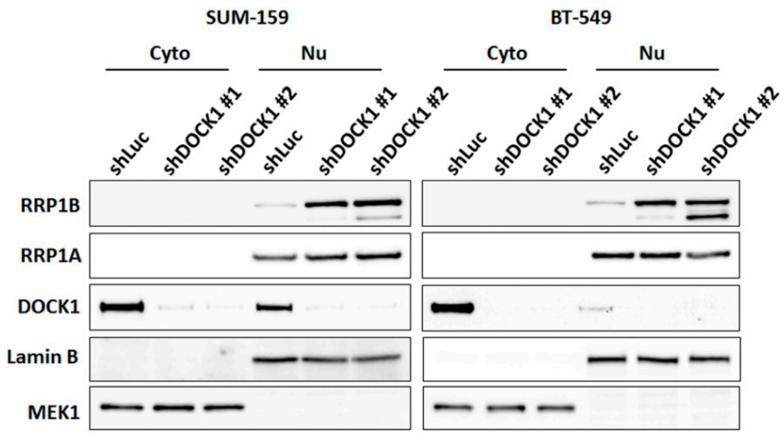

Figure S2. Knockdown of DOCK1 increases nuclear RRP1B levels. Claudin-low breast cancer cells were treated with shDOCK1 for 3 days. Cells were then harvested and fractionated into cytosolic and nuclear portions. Protein extracts from the fractions were used for protein determination Western blotting analysis. MEK1 and Lamin B served as a cytoplasmic (Cyto) and nuclear (Nu) control markers, respectively.

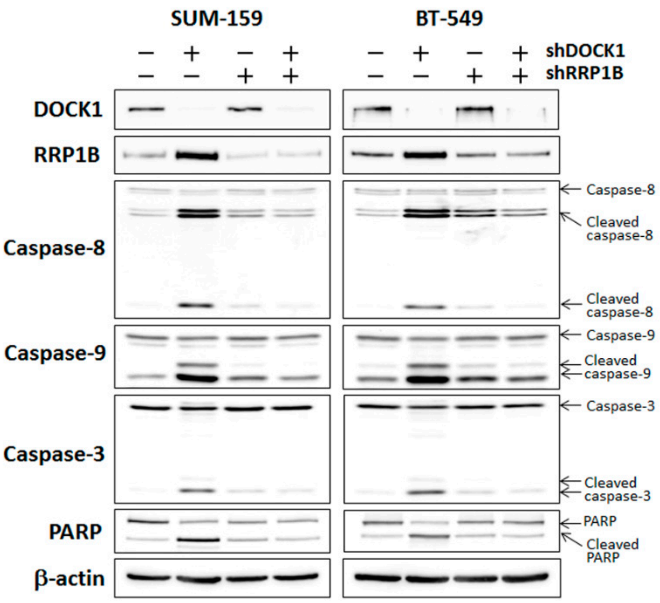

Figure S3. Knockdown of RRP1B attenuates caspase activation by DOCK1 depletion. Claudin-low breast cancer cells were treated with shDOCK1 and/or shRRP1B for 3 days. Cells were then harvested for protein abundance analysis by Western blot.

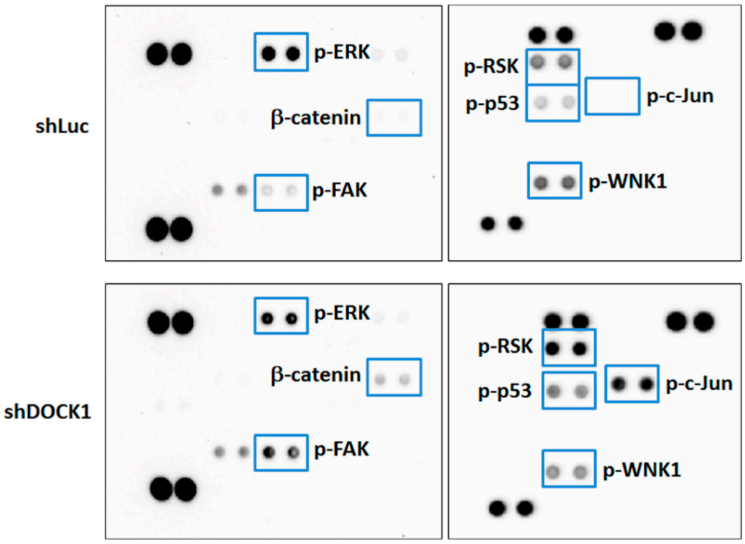

Figure S4. DOCK1 depletion alters the phosphorylation of specific protein kinases. MDA-MB-231 breast cancer cells were treated with shDOCK1 for 3 days. Cells were collected for protein phosphorylation analysis using a Human Phospho-Kinase Array (R&D Systems, Cat. #ARY003B).
